# The Eco-Exposome Concept: Supporting an Integrated Assessment of Mixtures of Environmental Chemicals

**DOI:** 10.1002/etc.5242

**Published:** 2022-01

**Authors:** Stefan Scholz, John W. Nichols, Beate I. Escher, Gerald T. Ankley, Rolf Altenburger, Brett Blackwell, Werner Brack, Lawrence Burkhard, Timothy W. Collette, Jon A. Doering, Drew Ekman, Kellie Fay, Fabian Fischer, Jörg Hackermüller, Joel C. Hoffman, Chih Lai, David Leuthold, Dalma Martinovic-Weigelt, Thorsten Reemtsma, Nathan Pollesch, Anthony Schroeder, Gerrit Schüürmann, Martin von Bergen

**Affiliations:** a Helmholtz Centre for Environmental Research—UFZ, Leipzig, Germany; b Office of Research and Development, Great Lakes Ecology and Toxicology Division, US Environmental Protection Agency, Duluth, Minnesota; c Environmental Toxicology, Center for Applied Geoscience, Eberhard Karls University Tubingen, Tubingen, Germany; d Institute for Environmental Research, Biologie V, RWTH Aachen University, Aachen, Germany; e Department of Evolutionary Ecology and Environmental Toxicology, Faculty of Biological Sciences, Goethe University Frankfurt, Frankfurt am Main, Germany; f Office of Research and Development, Ecosystem Processes Division, US Environmental Protection Agency, Athens, Georgia; g National Research Council, US Environmental Protection Agency, Duluth, Minnesota; h Office of Pollution Prevention and Toxics, Risk Assessment Division, US Environmental Protection Agency, Washington, DC; i College of Arts and Sciences, University of Saint Thomas, St. Paul, Minnesota, USA; j University of Minnesota Crookston, Crookston, Minnesota, USA; k Institute of Organic Chemistry, Technische Universitat Bergakademie Freiberg, Freiberg, Germany

**Keywords:** Exposome, Adverse outcome pathways, Suspect screening, Biomonitoring, Internal exposure, External exposure

## Abstract

Organisms are exposed to ever-changing complex mixtures of chemicals over the course of their lifetime. The need to more comprehensively describe this exposure and relate it to adverse health effects has led to formulation of the exposome concept in human toxicology. Whether this concept has utility in the context of environmental hazard and risk assessment has not been discussed in detail. In this Critical Perspective, we propose—by analogy to the human exposome—to define the eco-exposome as the totality of the internal exposure (anthropogenic and natural chemicals, their biotransformation products or adducts, and endogenous signaling molecules that may be sensitive to an anthropogenic chemical exposure) over the lifetime of an ecologically relevant organism. We describe how targeted and nontargeted chemical analyses and bioassays can be employed to characterize this exposure and discuss how the adverse outcome pathway concept could be used to link this exposure to adverse effects. Available methods, their limitations, and/or requirement for improvements for practical application of the eco-exposome concept are discussed. Even though analysis of the eco-exposome can be resource-intensive and challenging, new approaches and technologies make this assessment increasingly feasible. Furthermore, an improved understanding of mechanistic relationships between external chemical exposure(s), internal chemical exposure(s), and biological effects could result in the development of proxies, that is, relatively simple chemical and biological measurements that could be used to complement internal exposure assessment or infer the internal exposure when it is difficult to measure.

## INTRODUCTION

Organisms are exposed to thousands of chemicals from natural and anthropogenic origins throughout their lifetime. Typically, however, prospective assessments of chemical risk are based on characterizing potential adverse effects caused by single chemicals. Similarly, retrospective assessments designed to evaluate the efficacy of prospective assessments are usually limited to an analysis of a subset of chemicals to which an organism may be exposed. To fully understand the combined effects of chemicals (Kortenkamp & Faust, 2018), a comprehensive description of exposure and related environmental hazards is therefore crucial. Monitoring of exposure from the external environment (e.g., via media such as water or sediments) can be expanded to cover an ever larger number of chemicals, in part through the use of bioassays for a more integrated assessment (Altenburger et al., 2019). If the goal, however, is to predict adverse effects on organisms and populations, the composition and amounts of chemicals (or their transformation products) that are bioavailable to interact with internal molecular targets are most relevant. To better describe this internal exposure, human health researchers have developed the concept of a chemical “exposome,” which comprehensively describes the internal chemical environment. The ultimate goal of this approach is to provide a clearer linkage between chemical exposures and adverse effects, recognizing the complex interplay between chemical impacts and the organism’s responses to contaminant stressors (Escher et al., 2017; G. W. Miller & Jones, 2013; Peters et al., 2011; Rappaport, 2011, 2012; Wild, 2005, 2012).

Historically, ecotoxicology has largely focused on external exposure concentrations for deriving effect concentrations of concern. Starting in the mid-1980s, however, the concepts of critical body residues and lethal body burdens, with a focus on single chemicals, began to emphasize the relevance of internal concentration (or dose) as opposed to external exposure (Escher et al., 2011; Jarvinen & Ankley, 1999; McCarty et al., 2011; McCarty & Mackay, 1993; Meador et al., 2008). As for human health assessments, a comprehensive description of complex internal exposures rather than a focus on individual chemicals could be useful in both prospective and retrospective assessments of hazards to ensure that the risks of individual chemicals can be evaluated in the context of mixtures.

The objectives of this Critical Perspective are to provide a useful definition of the eco-exposome; elucidate the requirements, limitations, and challenges associated with assessment of the eco-exposome; and identify available experimental and computational tools for assessment of the eco-exposome. Recent developments in chemical analytics and biological effects assessment have made possible a more comprehensive description of chemical exposure in humans. The same technological advancements can be adopted in ecotoxicology. Therefore, it is timely to discuss the application of the exposome concept for environmental organisms.

The article is base on presentations and discussion emanating from a workshop coordinated by the US Environmental Protection Agency and the Helmholtz Centre for Environmetal Research - UFZ, Germany, held in August 2017 in Duluth, Minnesota, USA.

## DEFINITION OF THE ECO-EXPOSOME

The (human) exposome has been defined as the totality of exposure from conception to end of life, including exposures to exogenous chemicals and natural products, as well as chemicals generated internally in response to toxic insult or lifestyle factors such as diet, smoking, and stress (Rappaport, 2011; Wild, 2005, 2012). Recent applications of this approach include studies investigating the link between air pollution and coronary heart disease/asthma (Vineis et al., 2020), potential effects of air pollution on lung function in children (Agier et al., 2019), and the association between in utero chemical exposure and fetal growth (Agier et al., 2020).

The term “eco-exposome” was introduced in the National Research Council report *Exposure Science in the 21st Century: A Vision and Strategy* (National Research Council, 2012). In that report, the eco-exposome was defined as “the extension of exposure science from the point of contact between stressor and receptor inward into the organism and outward to the general environment, including the ecosphere” and included “narrating the flow and pulse of exposures through the ecosphere, of which humans are part, [promoting] … a more thorough investigation of the potential sources of exposure and how these sources can be controlled to protect public and ecosystem health.” In this definition, the environment was considered as a meta-organism, and the boundaries separating external and internal exposures were not clearly defined.

With the goal of harmonizing the assessment of human and environmental health risks, we propose a narrower definition of the eco-exposome that aligns with the human exposome definition. Our definition (see Textbox 1) expands on that previously proposed by Escher et al. (2017) and is focused on the internal concentration in a manner similar to that outlined for the assessment of pharmaceuticals in aquatic fauna (T. H. Miller et al., 2018). This focus on internal exposure is required to improve the linkage between exposure and effects because typically only chemicals that enter the organism contribute to disruption of cellular functions, potentially leading to adverse effects (Escher & Hermens, 2004).

Although the human exposome and eco-exposome are defined in similar terms, there are differences in the application of these concepts. Measurements of the human exposome are typically limited to nondestructive assessment of blood, urine, or feces. In contrast, measurements made in the context of the eco-exposome can use whole organisms or portions thereof, including known or suspected target tissues. In practice, individual susceptibility represents an important factor for translation of the internal exposure to effects in humans, whereas in ecotoxicology the assessment is most often focused on maintenance of self-sustaining populations rather than the health of individuals. With respect to application of the eco-exposome concept, many species (or model representatives of their taxa/trophic level) are available for small-scale and/or high-throughput screening studies, which enables experimental examination of mechanisms and perturbed pathways through which a given internal exposure leads to adverse outcomes. This direct verification of hypothesized adverse effects is only possible to a limited extent in humans (see, e.g., Preston et al., 2020). By comparison with the human exposome, integration of the eco-exposome over time can be more easily established by trans-sectional analysis; that is, by subsampling different life stages from an entire population (an example of flame retardants is described by Su et al. [2017]). Furthermore, for some ecologically relevant organisms, the life span is short enough that the eco-exposome can be observed during their entire lifetime. One major challenge of the eco-exposome concept involves the numerous taxonomic groups and species in an ecosystem; however, one could envisage using selected species as sentinels for a specific trophic level or environmental compartment, as is often done for ecological risk assessment (van Leeuwen & Vermeire, 2007).

## PRINCIPLES OF THE ECO-EXPOSOME ASSESSMENT

### Chemical versus bioanalytical assessment of the exposome

While initial assessments of the human exposome focused largely on chemical measurements, simultaneous consideration of the biological attributes of the internal environment may provide additional means to better characterize complex exposures. Accordingly, some authors have proposed that assessment of the (eco-)exposome should be complemented by the use of biochemical receptor-binding assays (Chung et al., 2021), in vitro cellular bioassays (Escher et al., 2017; Wild, 2012), and targeted omics techniques (Escher et al., 2017; Wild, 2012). Bioassays can be used to measure effects caused by extracted internal chemicals, thereby improving assessment of the eco-exposome. While chemical analytics can provide internal exposure information for thousands of exogenous compounds, current methods cannot detect all chemicals of potential toxicological concern, so concurrent measures of biological effects may help account for undetected yet toxicologically important chemicals and provide insight on potential mixture effects such as synergism or antagonism (Escher et al., 2020).

Bioanalytical assessment would also allow for improved consideration of the role of endogenous chemicals synthesized by the body in response to exogenous chemicals and relevant for signaling processes that trigger diverse toxicity pathways. For instance, exposure to exogenous chemicals can result in elevated levels of reactive oxygen species (ROS) normally produced by the body. Elevated ROS levels lead to covalent modification of a sensor protein and stabilization of the nuclear factor erythroid 2–related factor 2 transcription factor, resulting in induction of antioxidant response pathways (Yamamoto et al., 2018). While a meta-analysis revealed elevated ROS levels in organisms exposed to higher levels of some pollutants in the environment (Isaksson, 2010), it is unclear whether elevated ROS represents a biomarker of exposure or whether this response contributes to the internal exposure and may mediate adverse effects. Similarly, endogenous hormone levels may change in response to exposure to an environmental contaminant such as a sex steroid or thyroid hormone synthesis inhibitor. Here, there is strong evidence that a change in hormone levels, that is, the internal exposure, may represent a key causative event for the propagation of adverse effects (Conolly et al., 2017; Crofton, 2008). Over time, however, this adverse effect may trigger changes to the internal chemical environment (e.g., through compensatory feedback mechanisms), resulting in an even more complex situation, which is relevant to an integrated translation of exposure to biological effects. These types of complex scenarios may be resolved by combining (internal) chemical exposure assessment with analysis of key biological signaling molecules relevant to known or suspected toxicity pathways. (A change in signaling molecule abundance can also be defined as a key event [KE] in an adverse outcome pathway [AOP; see section Making the Link between Exposure and Effect]. In this Critical Perspective, we consider such changes to be part of a complex chemical exposure given that these molecules might be coextracted with exogenous chemicals [see section Extraction] and contribute to an internal chemical environment associated with toxic effects and organismal responses to such effects.)

**FIGURE 1:**
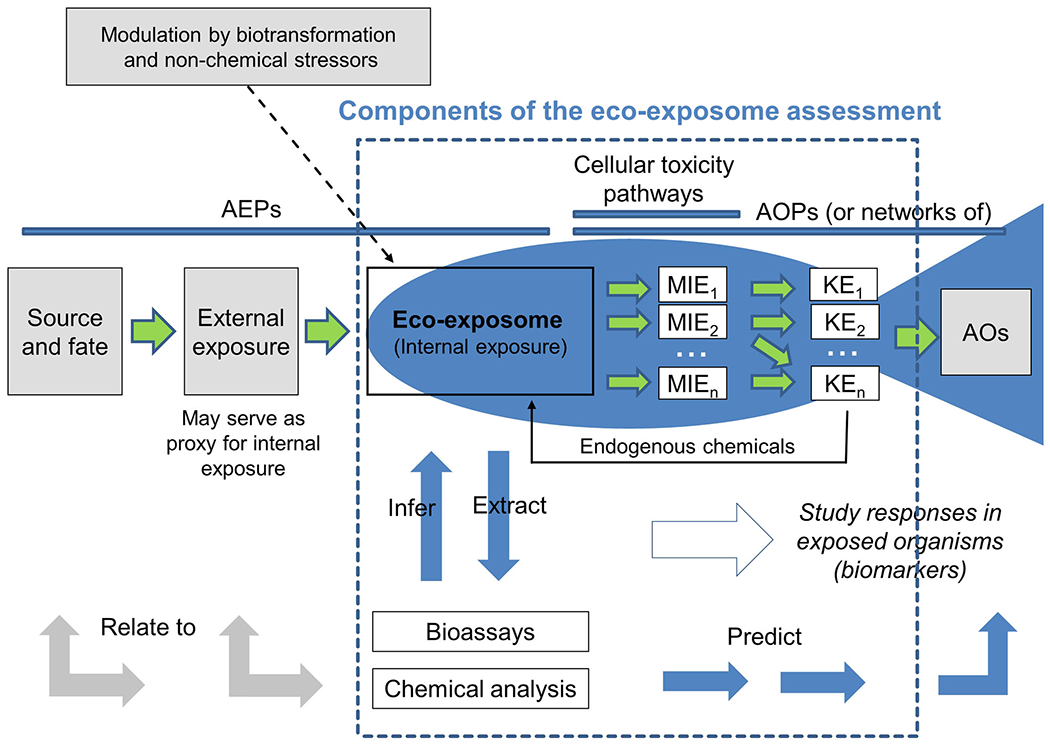
The eco-exposome assessment in relation to aggregate exposure pathways, cellular toxicity pathways, and adverse outcome pathways. The fish sketch represents one possible organism for which an eco-exposome assessment could be conducted. Endogenous chemicals are chemicals normally synthesized by the body that are involved in some type of cell signaling pathway. The levels of these endogenous chemicals may change as a direct consequence of exposure to exogenous chemicals and/or because of cellular and organismal responses to toxic effects. AEP = aggregate exposure pathway; AOP = adverse outcome pathway; MIE = molecular initiation event; KE = key event; AO = adverse outcome.

The complementary application of chemical analysis and bioassays requires information to link exposures to adverse effects of regulatory significance. Establishing these links can be achieved by using the AOP framework. The AOP concept was first described approximately a decade ago to support the translation of responses measured at different levels of biological organization into effects germane to environmental risk assessors, specifically changes in survival, growth/development, and reproduction that influence the status of populations of organisms (Ankley et al., 2010). An AOP describes the supporting evidence for an initial interaction of a chemical with a biological macromolecule (the molecular initiating event [MIE]; e.g., an enzyme, receptor, or DNA) and the subsequent downstream changes across levels of biological organization (captured as measurable KEs) that culminate in an apical response of concern, the adverse outcome (Figure 1). Hundreds of published studies have employed the AOP concept as a method to organize and interpret biological data in the context of predictive toxicology. For this reason, the AOP framework has received considerable international support relative to regulatory activities associated with chemical risk assessment (https://www.oecd.org/chemicalsafety/testing/projects-adverse-outcome-pathways.htm).

While the AOP concept has proven to be a valuable tool to support effects assessment, it is limited by not having an explicit consideration of chemical exposure and toxicokinetics upstream of the MIE. To address this gap, the aggregate exposure pathway (AEP) framework was proposed. An AEP describes the pathway from source to environmental media and external to internal concentrations. Hence, it complements and links to AOPs (Teeguarden et al., 2016; Figure 1). However, the AEP describes individual chemicals, or groups of chemicals, linked to a specific MIE as exemplified by perchlorate exposure and inhibition of the sodium iodine symporter (Hines et al., 2018). The eco-exposome concept provides a more holistic vision of chemical risk assessment that considers the interactions in complex mixtures, thereby augmenting the role of AEPs.

### Eco-exposome versus mixture risk assessment

A risk assessment of ecological impacts based on individual chemicals does not capture the reality of most environmental scenarios where exposure is to mixtures of chemicals (Kortenkamp & Faust, 2018). Historically, assessments of well-defined mixtures of toxicologically well-characterized chemicals have been performed and combined with predictive models for concentration addition (for chemicals with similar mode of action) or independent action (for chemicals with dissimilar action; Faust et al., 2003). A relevant example of the former approach involves polychlorinated biphenyls (PCBs), polychlorinated dibenzofurans, and polychlorinated dibenzo-*p*-dioxins, where potential mixture effects have been based on a common mechanism of action, activation of the aryl hydrocarbon receptor (Van den Berg et al., 1998). A mixture assessment can also be performed by summation of toxic units with or without consideration of the mode of action (Kienzler et al., 2016; Posthuma et al., 2019). The foregoing approaches to mixture assessment are restricted, however, to chemicals that are identified as mixture components, can be quantified, and for which appropriate toxicity data exist. For complex environmental mixtures, these traditional approaches would only apply to a portion of the chemicals detected by chemical analysis (Escher et al., 2020).

The eco-exposome concept seeks to comprehensively characterize the internal chemical environment, including endogenous signaling molecules that may be affected by exposure to exogenous chemicals. As such, the eco-exposome concept goes well beyond the traditional assessment of defined chemical mixtures. Because of this expanded mixture perspective, the eco-exposome assessment is potentially better suited to understanding biological effects in the context of simultaneous or sequential perturbations for multiple pathways/responses that derive from unknown components. Furthermore, by combining the eco-exposome assessment with knowledge of chemical exposure and the mode of action of chemicals of concern, a mechanistic link between complex exposure scenarios and effect may be established.

## METHODS TO MEASURE AND INTERPRET THE ECO-EXPOSOME

**FIGURE 2:**
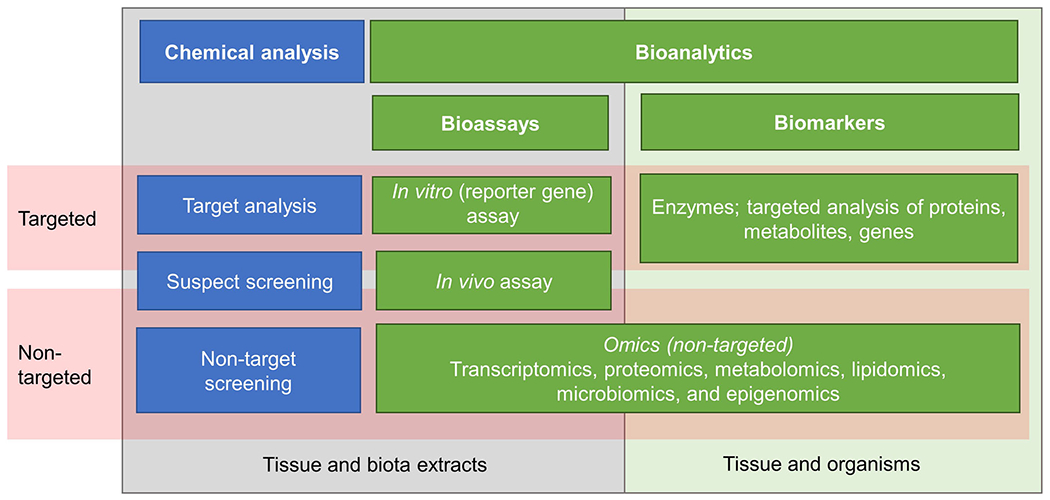
Analytical and effect-based methods to characterize the eco-exposome.

The goal of performing eco-exposome assessments is made possible by the availability of established methods for sampling, sample extraction, targeted and nontargeted chemical analyses and bioassays, and data integration and interpretation. In this section, we describe the nature and application of some of these basic tools (Figure 2). The focus is on principles for internal exposure assessment and, when available, on organisms in the environment. Methods for assessment of external exposure are considered when the approach may be adapted to internal exposure prediction/assessment. Given that eco-exposome analysis is a novel approach not yet routinely applied in environmental hazard and risk assessment, the available tools and feasibility of performing a comprehensive assessment may considerably change in the future.

### Sampling

An important initial step for eco-exposome assessment is a thorough design and planning of sample collection. The type of sample (e.g., whole animals vs. specific organs or body fluids) and the timing/season/frequency of sample collection, possibly including trans-sectional sampling of life stages or age groups, all dictate the type of information and associations that can be obtained through an eco-exposome assessment. To discriminate chemical from nonchemical stressors, information on confounding factors such as nutritional status, temperature stress, habitat quality, and so on, should be considered for the sampling design; and appropriate additional samples and data should be collected. Nondestructive approaches applied to humans, such as analysis of saliva or urine (Bessonneau et al., 2017; Maitre et al., 2018), usually would not be required for environmental assessments. However, in instances where these approaches might be required and possible (e.g., threatened or endangered species, large species), noninvasive sampling techniques may be useful. Examples include analysis of fish mucus and feces (Ekman et al., 2015; Hano et al., 2018).

### Extraction

An array of methods is available to extract chemicals from biological tissues; however, none of these methods are comprehensive in terms of analyte coverage. It is common, therefore, to tailor individual methods to specific chemical classes (e.g., metals vs. organics, hydrophilic vs. hydrophobic chemicals). Furthermore, exhaustive extracts may contain many endogenous chemicals, so sample cleanup is often required to avoid damaging analytical instrumentation and/or confounding effects (e.g., masking effects; see Table 1) in chemical analysis and bioassays (Reiter et al., 2020). For direct comparison of chemical analysis and bioassay results, use of the same extraction and cleanup methods (albeit not necessarily the same sample) is ideal.

Tissues are typically extracted using organic solvents, followed by different cleanup steps, often focused on the digestion or removal of coextracted lipids or other matrix constituents (Baduel et al., 2015). Such cleanup steps need to be adapted to the analytes of interest, to avoid their removal and/or degradation (Schlittenbauer et al., 2015). This is especially challenging if the methods aim at detecting a wide range of analytes including both exogenous chemicals and biogenic molecules, such as in nontarget screening (Baduel et al., 2015). Equilibrium passive sampling with polymers such as silicone can be used as an alternative extraction technique for neutral, low–molecular weight chemicals covering a wide range of hydrophobicity, with octanol–water partition coefficients from approximately 10 to 10^8^. Depending on the size of an organism, passive samplings can be applied to the whole organism, homogenized tissues, or body fluids (Jahnke et al., 2014; Jin et al., 2013; Jin, Escher, et al., 2015; Rojo-Nieto et al., 2019). Extraction with polymers avoids or largely reduces coextraction of the matrix because proteins and ions do not partition into polymers. Therefore, passive sampling extracts can be subjected to chemical analysis or bioassays of the resulting samples without further cleanup.

While it could be interesting—and relevant—to consider different organs (e.g., liver, brain) or fluids (e.g., plasma, urine) when characterizing internal exposures, this is difficult for many ecologically relevant organisms because of their small body size (e.g., invertebrates, young animals, and small fish species). In these cases, whole-body extractions may have to be used. Higher levels in specific organs, however, could be diluted by whole-body extractions. In some cases, toxicokinetic (TK) models developed for larger species may be used to extrapolate from whole-body to tissue-specific chemical concentrations. Alternatively, chemical analytics combined with spatial imaging could be applied for small species (Halbach et al., 2019; Kirla et al., 2016).

### Chemical analysis

Chemical analysis methods range from targeted methods that are based on the use of standard compounds to suspect screening of expected chemicals such as predicted metabolites without available reference standards to nontarget methods that aim to detect and identify chemicals unknown from the perspective of described chemical structure, chromatographic properties, and fragmentation patterns. Similar to extraction methods, the choices made in designing and optimizing analytical methods will impact the suite of chemicals that can be detected in both targeted and nontargeted analyses. No single method will be truly comprehensive. For instance, the choice of the chromatographic method, the ionization technique, or the use of gas chromatography versus liquid chromatography for mass spectronomy analyses will limit detection to compounds with amenable chromatographic properties. However, some of these limitations could be overcome by combining different methods.

#### Targeted chemical analysis.

There are thousands of chemicals that could occur in a given environmental sample. However, by necessity, chemical risk assessments typically focus only on relatively few of these, defined by the overlapping subset of what is perceived to be of concern in terms of unacceptable biological effects and what can actually be measured. In tissues, analytical methods often focus on chemicals with high persistence (e.g., organic pollutants such as PCBs, dioxins) and/or known/anticipated biological activity (e.g., polycyclic aromatic hydrocarbons, pesticides, some pharmaceuticals; T. H. Miller et al., 2018). Persistent organic pollutants have been focused on in the past because of their high hazard potential.

Targeted approaches have the advantage that extraction and detection can be tailored for the chemicals(s) of interest, providing maximum specificity, sensitivity, and data quality. In contrast, if a large number of chemicals is to be detected simultaneously (or if nontargeted analysis is conducted, see section Nontargeted Chemical Analysis), the analytical protocols have to compromise toward the detection of a large variety of chemicals. An additional limitation of targeted analysis is, of course, the need to have a standard for each of the targeted chemicals.

#### Suspect screening analysis.

Suspect screening is used to extend chemical screening beyond a limited number of targets to a set of possibly or likely occurring compounds even if no reference standards are available. To this end, chromatograms are screened with high-resolution mass spectrometry (HRMS) for large sets of compounds with known or predicted structures and molecular masses of concern. Suspect screening can be based on chemical suspect lists such as that available through the NORMAN Suspect List Exchange (https://www.norman-network.com/nds/SLE/). Alternatively, suspect screening can be focused on suspects closely related to the study aim such as predicted metabolites of target compounds (Krauss et al., 2010). Suspect screening has been widely applied for the analysis of water and sediment samples (e.g., Chiaia-Hernandez et al., 2014; Hug et al., 2014). Relatively fewer examples exist for application of this method to body fluids (e.g., human urine or blood [Plassmann, Brack, et al., 2015; Plassmann, Schmidt, et al., 2015]) or tissue extracts of environmental organisms (Du et al., 2017; Musatadi et al., 2020).

#### Nontargeted chemical analysis.

Targeted approaches, even when they detect a large number and variety of chemicals, cannot detect all chemicals of potential toxicological relevance. Recognizing this fact, there has been an increasing emphasis on development of nontargeted analytical approaches for complex mixture assessment that are more comprehensive in terms of coverage and not restricted to chemicals for which analytical standards are available (Hollender et al., 2017; Schymanski et al., 2015). Nontarget screening employs HRMS to determine the mass of molecular ions of chemicals as accurately as possible. The overwhelming number of signals for which information is limited typically demands these signals be prioritized prior to their identification. This prioritization depends on the aim of the study and may address frequent or rare peaks, peaks with higher intensities, or those occurring only in affected organisms. Identification of some chemicals can be achieved using databases, such as Wiley EI-MS, NIST-EI-MS, or METLIN (Milman, 2015). The US Environmental Protection Agency’s chemistry dashboard—which provides information for more than 700 000 compounds—also can be used to identify chemical structures in nontargeted HRMS screening studies (McEachran et al., 2018). Identification of detected chemicals is critical to establishing plausible linkages to possible biological effects and move from qualitative non-target screening to a quantitative assessment. However, even when the nontargeted analysis by HRMS does not reveal compound identity, chemical “fingerprints” consisting of the totality of (nonidentified) signals may provide insights as to exposure sources and trigger suspect screening for these fingerprints in the exposure pathway from source to biological tissues (Brack et al., 2019). To date, non-targeted analytical approaches have rarely been applied to extracts of environmental species. One recent example is the analysis of contaminants in northern pike, which identified the plasticizer diethyl phthalate and the surfactant perfluorooctane sulfonic acid among the compounds with relatively high abundance (Tian et al., 2019). Another example was provided by reanalysis of full-scan HRMS data from fish fillet samples previously subjected to targeted analysis. This nontargeted assessment led to identification of additional perfluoralkyl substances, including polyfluorinated carboxylic acids and polyfluorinated telomer alcohols, hydroxylated polychlorinated biphenyls, and various pesticides, herbicides, antifungals, pharmaceuticals, artificial sweeteners, and personal care products (Baygi et al., 2021).

#### Chemical adducts.

Electrophilic chemicals can form covalent bonds with DNA, proteins, or glutathione, which requires a different approach for analysis compared with solvent-based extraction of nonbound chemicals. As with unbound chemicals, protocols are available for targeted as well as nontargeted analyses. The latter protocols are used for assessment of the adductome, which represents the totality of chemicals bound to tissues or nucleophiles (Rappaport et al., 2012). Analysis of DNA adducts generally requires enzymatic digestion of a sample followed by a cleanup step to enrich the adducts before application of MS-based methods (Balbo et al., 2014). In the case of proteins, the assessment is typically focused on selected molecules such as serum albumin or hemoglobin (Rappaport et al., 2012). Proteins with covalent modifications are enriched, digested, and subsequently analyzed with MS.

### Bioanalytics

Two scenarios for bioanalytical assessment of the eco-exposome are envisioned (Figures 1 and 2). In the first, bio-analytical tools such as in vitro assays would be employed to measure the activity of extracts as a complement to chemical analyses. Quantification of estrogenic effects or dioxin-like activities of mixtures extracted from tissue or blood using in vitro bioassays would represent examples of this approach (Erdmann et al., 2013). In the second scenario, effect-based methods would directly measure the consequences of exposure in the species of concern, such as changes in gene or protein expression, enzyme activity, or endogenous metabolite abundance linked to a specific toxicity pathway or AOP. The traditional (effect-) biomarker approach reflects this second approach, which integrates exposure and susceptibility to effects. Again, estrogenic chemicals represent an appropriate example; thus, induction of the egg yolk protein vitellogenin in male fish provides an integrated measure of internal exposure to chemicals that stimulate the estrogen receptor (Cavallin et al., 2016). For both scenarios, there are targeted and nontargeted biological approaches available (Figure 2).

#### Targeted bioanalytical assessment.

Targeted approaches may be used to study a narrow group of chemicals that interact with a specific molecular target (e.g., activation of the estrogen receptor) or a wide range of chemicals that provoke the same generalized response to a toxic insult (e.g., markers of oxidative stress). Ideally, targeted bioanalytical methods should be linked to an effect that is considered adverse and relevant for maintenance of animal populations. There are a number of examples where such bioanalytical methods have been applied to extracts of tissues or body fluids (Jin et al., 2015b), such as the assessment of estrogenic chemicals in dolphin blubber (Yordy et al., 2010), androgenic chemicals in the liver of various wild animals (Misaki et al., 2015) and dugongs (Jin et al., 2013), aryl hydrocarbon receptor agonists in fish and seafood (Kojima et al., 2011), thyroid hormone disruptors in polar bear blubber (Simon et al., 2013), immunotoxicants in blubber from polar bears and whales (Desforges et al., 2017), and chemicals in dugong blubber that induce adaptive stress response pathways (Jin et al., 2015a). Similar assays also have been used for the comparative assessment of surface waters (Blackwell et al., 2018; Neale et al., 2017; Schroeder et al., 2016). In theory, it is possible to use these methods to compare effects associated with chemical mixtures in water and biota; however, this approach would require that extraction procedures used for each sample matrix do not affect chemical composition or alternatively that effects on chemical composition are similar for both sample types. When these types of bioassays are applied as suites of measurements designed to capture multiple pathway-based bioactivities, the assessment becomes more comprehensive and closer to a nontargeted approach. Polymerase chain reaction arrays, targeted sequencing, and targeted MS-based proteome or metabolome analyses that focus on a diverse set of selected targets or certain groups of targets (Shi et al., 2016; Roberts et al., 2012; Kurokawa et al., 2004; Harrill et al., 2021) would represent approaches that are intermediate to traditional targeted and nontargeted assessments.

#### Nontargeted biological assessment.

There are several omics-based approaches that can be employed to comprehensively capture system-wide responses without preselection of specific endpoints (Figure 2). Observed changes at the molecular level not only capture integrated responses to contaminant mixtures but potentially can be linked to adverse effects through application of the AOP framework and associated concepts (Brockmeier et al., 2017). This linkage to adverse effects can be supported by analyzing the enrichment of predefined pathway-specific gene sets or AOP-related gene sets (Subramanian et al., 2005) based on curated databases such as the Kyoto Encyclopedia of Genes and Genomes (Kanehisa & Goto, 2000) or the Comparative Toxicogenomics Database (CTD). The latter database represents an assembly of gene expression data from hundreds of different studies with chemical stressors (Davis et al., 2019). One challenge in establishing causative linkages of chemicals to adverse effects is that it can be difficult to distinguish primary, direct effects from indirect compensatory and adaptive biological responses. In the future, association of measured gene/metabolite responses with KEs represented in AOPs may facilitate the distinction of direct effects responsible for adverse outcomes (Pittman et al., 2018).

A specific benefit of metabolomic analyses is that the MS-based techniques employed can measure endogenous and exogenous chemicals, thereby providing an integrated data set that links internal exposure to potential effects (Niedzwiecki, 2019). In addition, these analyses are generally performed on biofluids such as urine and bile, which provides potentially important information regarding the metabolism of both endogenously and exogenously derived chemicals (Bouatra et al., 2013).

Individual omics-based techniques may only show responses to specific classes of biomolecules. Ideally, therefore, a multi-omics approach should be employed (see Canzler et al., 2020). Omics-based approaches can be applied directly to an organism sampled from a contaminated environment. Alternatively, extracts of tissues from an organism could be used to measure omics responses using in vitro systems. To date, however, this second approach has only been applied to organisms or cells that were exposed to extracts from water rather than tissue extracts. For example, Zhen et al. (2018) described the use of metabolomics in a zebrafish cell line treated with water extracts containing complex chemical mixtures to define biological pathways perturbed by components of the sample. While such an approach can support initial identification of potential hazards associated with a given exposure scenario, additional investigation is required to establish whether a specific risk is actually relevant to a species of concern in the environment under consideration.

### Challenges, confounding factors, and limitations for assessment of the eco-exposome

Despite the technological advances we have described, there are several challenges associated with an eco-exposome assessment (Table 1), including the analytical discrimination of exogenous chemicals from endogenous chemicals present at very high concentrations. This challenge may hamper efforts to link observed effects to toxic (but unrecognized) chemicals present as part of a larger mixture. In such cases, an external exposure assessment (i.e., measurement of these chemicals in environmental media) may serve as an indicator of potential internal exposure. A key feature of an eco-exposome assessment is the goal of integrating an organism’s cumulative exposure over its entire lifetime. In particular, the capture of critical time windows of life stage–specific susceptibility may be important to link the eco-exposome with adverse effects. In some cases, it may be sufficient to apply a trans-sectional assessment, which involves simultaneous monitoring of many organisms in a given population at different life stages. In addition, there may be cases where the exposure is relatively stable because of the size of the receiving water (e.g., in large lakes) or because of a continuous chemical discharge (e.g., observed for the river Danube [Rico et al., 2016]). In such instances, it would be feasible to conduct an exposure assessment over an entire lifetime for organisms that have a short life span. In cases of intermittent exposure, a complexity may be approached that is difficult to resolve. These intermittent exposures can be further complicated by life-history factors such as migration and reproduction. Finally, species-specific factors such as habitat preference can impact any type of exposure, making it difficult to extrapolate between species. In general, integration of ecological factors into the exposome is challenging but could be achieved through various approaches. For instance, ecological parameters could serve as modulating factors when predicting the transition from exposure to population effects using the AOP framework (see section Making the Link between Exposure and Effect). Such an attempt has been suggested to estimate potential combined effects of climate change and chemical exposure and may be extended to an exposome assessment (Hooper et al., 2013). Approaches that used Bayesian network models represent a further example to estimate the impact of chemical exposure versus other environmental factors (e.g., oxygen levels, temperature, habitat, population structure; Mitchell et al., 2021) with a focus on population development and could be applied in the exposome assessment as well.

Assessment of the eco-exposome inherently accounts for TK processes that control chemical uptake and accumulation in exposed organisms. It does not resolve these TK processes; however, an understanding of TK would be relevant for translating an internal exposure to an external exposure (“reverse toxicokinetics”) and could provide a basis for extrapolating internal exposure data to untested species. The TK models used to perform these extrapolations can be informed by in vitro measurement of key parameters such as plasma protein binding and intrinsic metabolic clearance (Pearce et al., 2017). For species extrapolation of effects, toxicodynamic differences between organisms may be important. For example, cross-species conservation of relevant protein targets for a given chemical and the subsequent chain of events leading to a potential adverse effect can vary among species. Computational tools to evaluate molecular target similarity such as SeqAPASS (Sequence Alignment to Predict across Species Susceptibility) could be used to assess cross-species susceptibility to chemicals of concern (LaLone et al., 2016).

Information pertaining to nutritional status, behavior, and duration/fluctuations in exposure may also be important for cross-species extrapolation of exposure and effects. Tracers of diet and habitat (e.g., carbon and nitrogen stable isotope ratios, fatty acids) have been used to assess dietary exposures to chemical stressors and examine risk relative to population development (Bracey et al., 2020; Cabana & Rasmussen, 1994; Hoffman et al., 2020). These studies show that diet, trophic level, habitat, and behavior (movement) can vary by life stage, resulting in stage- or age-specific changes in both individual exposure and translation of external exposure to internal concentration. These stage- or age-specific changes result in substantial interpopulation variability in risk, especially when integrated over an animal’s lifetime. These tracers may also be applied to estimate the contribution of nonchemical stressors such as the presence of invasive species which can affect diet, nutrition, or habitat (Lepak et al., 2019). Finally, because the ultimate goal of environmental risk assessment is the protection of populations and ecosystems, a translation from the individual level is required. This is also a major difference relative to the focus of human exposome assessments on individual health, which requires additional efforts to address the population relevance. The translation from the individual to the population is a challenging task and cannot be experimentally verified easily. Therefore, at least for a screening-level analysis, this might be achieved using computational tools to predict population responses (Kramer et al., 2011; Perkins et al., 2019).

While some of the limitations described in this Critical Perspectives are challenging to overcome, practical application of the eco-exposome concept nonetheless enables development of a formalized framework for defining plausible linkages between exposure and effects. At present, it is somewhat difficult to apply experience gained from human exposome assessments because many of these have been hypothesis-driven and/or restricted to the assessment of a fairly limited (<100) number of analytes (Huhn et al., 2021).

## MAKING THE LINK BETWEEN EXPOSURE AND EFFECT

The goal of a comprehensive exposure assessment is to provide a basis for diagnosis of chemicals or groups of chemicals to which organisms were exposed and/or reliable and quantitative prediction of adverse effects. A variety of agnostic (i.e., nontarget) statistical and computational approaches, including logistic regression, partial least squares regression (PLS), and machine learning, have been utilized to identify environmental factors associated with biological effects in both eco- and human exposome research (Collette et al., 2019; Jeong et al., 2018; Rappaport, 2012; Zheng et al., 2020). For example, Collette et al. (2019) used PLS combined with cross-validated predictive residuals analyses to identify stressors related to endogenous metabolite profiles in a zebrafish cell line exposed to water sample extracts from 38 different US streams. A comparative assessment of analytical, regression-based approaches for exposome studies indicated multivariate approaches (e.g., sparse PLS, Graphical Unit Evolutionary Stochastic Search) as preferable to univariate approaches (Agier et al., 2016). Alternatively, the Connectivity Map approach applied to relate omics responses in fish to chemicals in water samples could be applied to establish links to internal concentrations (Wang et al., 2016). Ontology-based analysis of effect patterns (Wang et al., 2019) or machine learning approaches (Nagata et al., 2014) established in other scientific fields also could be adapted to discover associations in an eco-exposome context.

Several studies have integrated prior knowledge of chemical-biological interactions (e.g., genes, metabolites) to link external exposures to observed effects on the transcriptome using information from databases such as CTD (Schroeder et al., 2017; Williams et al., 2011). Given the absence of standardized data analytics approaches and a bias of a priori methods toward known exposure–effect associations, an integration of multiple lines of evidence stemming from agnostic and a priori knowledge-derived analyses may be the best available option. A study by Perkins et al. (2017) demonstrated how context likelihood of relatedness analysis can be integrated with a priori knowledge of chemical–gene interaction data to identify chemicals of concern. Another example of an integrative approach is the “meet in the middle” analysis deployed by a human exposome study where ultra-fine particle exposure and asthma were linked to several perturbed metabolic pathways, in part via logistic regression (Jeong et al., 2018).

**FIGURE 3:**
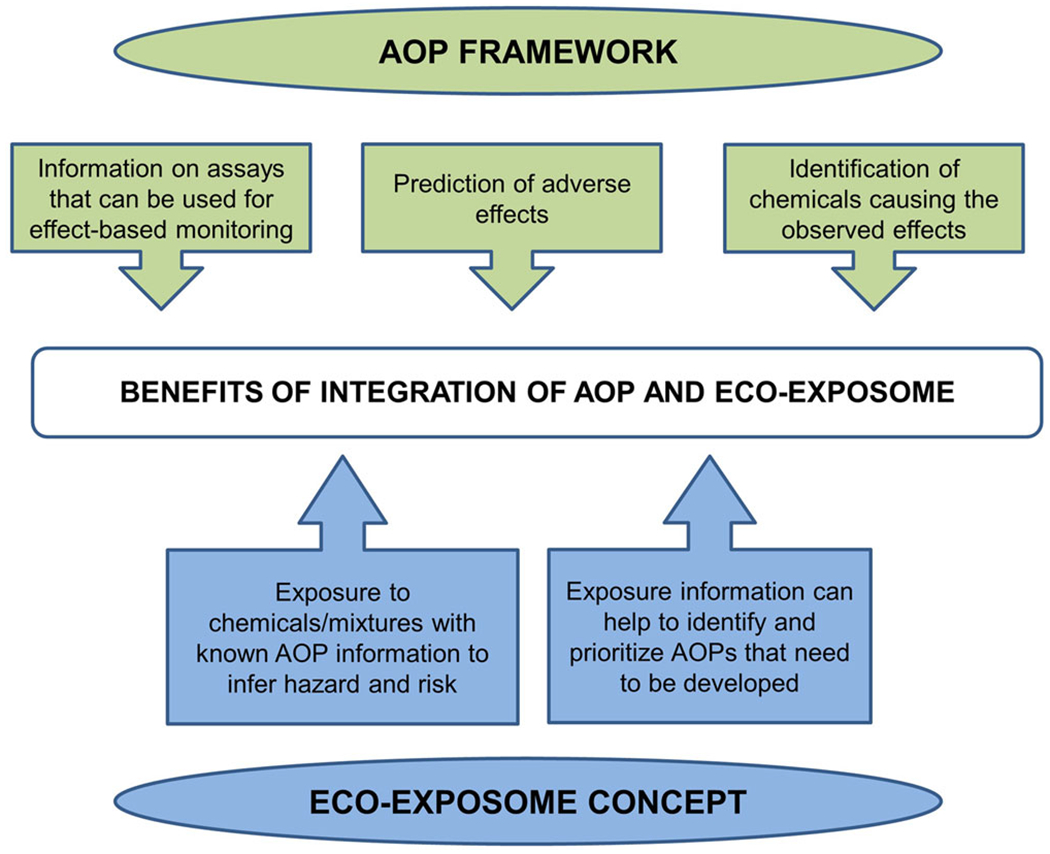
Benefits of the integration of the eco-exposome concept and the adverse outcome pathway framework. AOP = adverse outcome pathway.

While agnostic approaches are appropriate for identifying potential associations between exposure and effects, the establishment of causal links typically requires the incorporation of existing or new mechanistic information. The AOP framework is designed to facilitate the establishment of causal relationships between endpoints based on plausible relationships examined using weight-of evidence criteria (Becker et al., 2015). As such, AOPs are uniquely suited for identifying and supporting a mechanism-based link between chemical exposure and adverse effect(s). Eco-exposome assessments can benefit from integration of the AOP concept in at least two ways (Figure 3). First, AOPs such as those cataloged in the open-source AOP wiki (https://aopwiki.org/) can be used to predict potential adverse effects based on chemical/bioanalytical information obtained as part of the assessment. Second, information from AOPs can be used to identify relevant bioanalytical measurements that might be needed to address apical endpoints of potential concern in a given eco-exposome assessment scenario. For example, if an eco-exposome analysis is aimed at resolving potential reproductive effects in fish, there are several gene expression and/or metabolomic endpoints relevant to estrogen or androgen signaling or steroid hormone synthesis (Ankley et al., 2009). Conversely, eco-exposome measurements could be used to inform the development of AOPs. Specifically, the analytic portion of an eco-exposome analysis may indicate the presence of chemical stressors (or bioactivities) of concern, leading to targeted prioritization of AOP development based on the identification of relevant MIEs.

A major challenge associated with integration of the AOP and eco-exposome concepts is the need to address chemical mixtures that include individual components acting through known and unknown MIEs (and corresponding AOPs). To address this challenge, recent efforts have focused on the development and use of interactive AOP networks as a basis for predicting mixture effects (Knapen et al., 2018; Villeneuve et al., 2018). These efforts, while in their early stages, nonetheless offer a conceptual basis needed to apply pathway-based approaches to the prediction of toxicity associated with complex chemical mixtures (e.g., Ankley et al., 2020). Databases that link pathway information and molecular targets to MIEs and KEs may be used to infer known AOPs from omics data (Pittman et al., 2018). Conversely, toxicogenomic assessments may generate new hypotheses for AOPs targeted by an internal exposure and lead to the further development or application of targeted assays that support exposure assessment (Figure 2).

In principle, linked AOP-exposome assessments could provide a basis for tracing back from MIEs or downstream KEs to specific chemical stressors of greatest concern from an effects perspective. Examples of this type of retrospective analysis in environmental surveillance have been provided by Schroeder et al. (2016) and Corsi et al. (2019), albeit for external exposure assessment. Bioinformatic approaches such as the AOP Explorer, Bayesian network analysis, and other network-based tools can facilitate this type of decoding the eco-exposome for both the prediction of adverse effects and the diagnosis of exposure (Knapen et al., 2018; Perkins et al., 2019; Villeneuve et al., 2018).

An additional complicating factor in establishing links between exposure and effects is the time-varying nature of internal exposures. Furthermore, adaptation and recovery may be possible across a wide range of exposures, while exposures that exceed a critical threshold or persist for a sufficiently long period of time may overwhelm cellular (e.g., damage repair) and systemic physiological (e.g., homeostatic) defense mechanisms. Quantitative AOP models could be used to account for biochemical and physiological adaptations mediated by feedback loops, as illustrated by an example for steroid synthesis inhibitors and their effects on reproduction in fish (Conolly et al., 2017; Doering et al., 2019).

## SUMMARY AND PROSPECTUS

Among the greatest challenges facing toxicologists is the need to comprehensively assess exposure and establish causal linkages between this exposure and potential or observed biological changes. To assist in predicting risks associated with these exposures, toxicologists in the human health community proposed the concept of the exposome. While basing estimates of risk on some measure of an organism’s lifelong internal exposure to chemicals is intuitively appealing—and certainly reasonable from a toxicological perspective—it is emerging technologies in the areas of analytical chemistry, molecular biology, and informatics that have made assessment of the exposome a truly practical notion.

Incorporation of the exposome concept into ecological risk assessments is a logical evolution. There are, however, important differences between human health and ecological assessments with respect to the collection and interpretation of exposome data. Ecologically relevant species of concern may be very short-lived and provide comparatively small sample masses (relative to humans), which can result in the need for innovative sampling approaches such as the pooling of organisms and collection over a longer time period. In other instances, collection of eco-exposome data may be easier than for human health analyses; for example, for most species of interest it is possible to employ terminal (destructive) sample collection in both field and laboratory settings. In contrast, human biomonitoring studies are often based on the collection of body fluids such as blood or urine, which are not necessarily representative of the complete internal exposome.

Interpretation of exposome data presents different challenges for ecological versus human health assessments. For example, in ecological risk assessments, there is an emphasis on interpretation of exposure information in the context of population- rather than individual-level responses. In this Critical Perspective we have emphasized the complementary use of chemical analyses and bioassays as a means of evaluating the presence and integrated bioactivity of multiple contaminants operating via common or different toxicity pathways and capturing molecular and biochemical responses to an internal contaminant exposure. By incorporating molecular and biochemical endpoints into an eco-exposome assessment, it may be possible to “flag” the possible occurrence of undetected but biologically active chemicals. Such data also provide critical information required to interpret potential adverse effects on organisms of interest. Ultimately, the need to understand and predict risks associated with complex chemical mixtures requires a means to relate the resulting exposure (i.e., the exposome) to apical effects of interest to risk assessors. The AOP framework is uniquely well suited for this purpose.

Although the eco-exposome concept is itself relatively new, the basic approaches it encompasses are not. For example, ecotoxicologists have for many years emphasized the utility and importance of using internal chemical dose as a basis for predicting toxic effects. We are confident that as the eco-exposome concept is incorporated into assessment scenarios ranging from environmental monitoring to quantitative predictions of risk, the challenge of addressing the ecological effects of complex mixtures will become increasingly tractable.

## Figures and Tables

**TABLE 1:** Challenges and potential solutions for the eco-exposome assessment

Challenge	Description	Possible solutions
Comprehensiveness of chemical exposure assessment	In many cases, the identity of internal xenobiotic chemicals will be only partially known, hampering a comprehensive assessment because of methodological limitations such as lack of analytical standards, insensitivity of detection as a result of matrix effects, and masking of exogenous chemicals by endogenous chemicals (see “Endogenous vs. exogenous chemicals”).	Suspect screening may reveal the identity of more compounds of toxicological relevance. Matrix or masking effects from ions or macromolecules such as proteins may be avoided by using passive samplers to extract tissues or biofluids. Alternatively, proxies (e.g., external exposure assessment) may be used to infer internal exposure.
Endogenous versus exogenous compounds	Extraction of a biofluid, tissue, or whole-organism sample will yield both endogenous and exogenous compounds. The large number and often higher concentrations of endogenous compounds may mask the detection of compounds that have been taken up from the environment. This can be challenging for untargeted chemical analysis and may also influence results from bioanalytical assessment.	The use of surrogate environmental matrices or passive samplers (mimicking organisms) could help to differentiate between endogenous and exogenous compounds. By comparing sample extracts to extracts obtained from unexposed control organisms it may be possible to identify compounds of exogenous origin.
Formation of adducts	Reactive xenobiotic electrophiles can form adducts with biological macromolecules (e.g., DNA, proteins). These adducts are not captured by standard extraction protocols but are highly relevant with respect to biological responses (e.g., cancer, cytotoxicity).	Specific protocols are available to identify DNA or protein adducts and could be applied to complement standard extraction procedures.
Organ-specific assessment	It may not be feasible to analyze all relevant tissues in an organism. Furthermore, the assessment of internal concentrations in small organisms may only be possible in whole-body extracts.	Experience may indicate organs for which major differences in internal exposure can be expected, informing subsequent tissue sampling efforts. For small organisms, chemical imaging may provide information on tissue distribution
Exposure over lifetime	Albeit to a lesser degree than for external exposure, an individual sample taken for an exposome assessment represents a snapshot. Hence, exposure over the lifetime of an organism may be difficult to characterize if a limited number of “snapshot” samples are available. This would apply particularly to nonpersistent and/or hydrophilic chemicals, which may be rapidly excreted from an organism during recovery periods.	For organisms that have relatively short life cycles, fluctuations in external exposure over a relatively large time span may be less relevant than for long-lived organisms. Lipid-rich tissues may be useful for evaluating exposures to hydrophobic chemicals because these chemicals tend to remain in such tissues for a long period of time. Other chemicals with high persistence such as perfluoroalkyl substances may also be detected from snapshot samples in case of fluctuating exposures. Proper attention to sampling design and properties of targeted chemicals may increase the likelihood that limited samples adequately capture the nature of the exposure (see “Translation of the exposure event”).
Life-stage specificity	Organisms undergo complex transitions in their development, passing through windows of sensitivity with particular vulnerability to adverse toxicological effects. Exposure would only be relevant if it was present during such periods, and hence exposure and effect data may not match.	Trans-sectional sampling from populations could partially address this issue.
Species specificity	An exposome assessment inherently accounts for species differences in TK. Knowledge of TK could, however, be relevant for extrapolation between species. Current practice is the collection of data only for a limited number of species.	If the TK of a given chemical and organism are well understood, the toxicodynamics are conserved, and an appropriate TK model is available, extrapolation of chemical kinetics and effects from a tested species to untested species may be possible.
Modulation of internal exposure by diet, trophic level, the microbiome, parasites, habitat, and behavior (migration)	Diet, trophic level, the microbiome, habitat, parasites, and behavior can impact the translation of external exposures to internal concentrations. Diet and the microbiome may also impact the biotransformation of both endogenous and exogenous chemicals. Diet can be influenced by population structure, which may regulate access to food and exposure to certain chemicals.	These factors would be reflected in the assessment of internal exposure, but their individual impacts on the exposure would be unknown. For data interpretation and extrapolation among different species and environmental settings it would be useful to understand the impact of modulating factors on internal concentrations of exogenous chemicals. Controlled experiments could be conducted to study these impacts.
Endogenous chemicals changed by nonchemical stressors	Climate, nutrition, and habitat represent factors that may impact the levels of endogenous signaling chemicals and could confound identification of effects caused by exposure to exogenous chemicals.	Controlled experiments that study the impact of climate, nutrition, or habitat could help to identify changes in endogenous chemical levels unlikely to be caused by exposure to exogenous chemicals. Furthermore, tracers of diet and habitat (e.g., stable isotopes or fatty acids) may reveal changes in endogenous chemicals related to nonchemical stressors.
Translation of the exposure event	Temporal variations in exposure (e.g., peak runoff events vs. exposure to continuous discharge) may be differentially translated to internal concentrations, depending on the chemicals of interest. Particularly, peak exposure may be difficult to detect for nonpersistent compounds if samples are obtained outside of an ongoing discharge/event.	Sampling test species at different times and locations corresponding to different exposure situations would allow for a better resolution of fluctuating exposure.
Deriving population effects	The eco-exposome is based on an assessment of individual organisms. For environmental risk assessment, exposures and effects experienced at the individual level would have to be translated to the population level.	Translation of effects from individuals to populations is not a specific requirement in the eco-exposome assessment and is already part of the regulation of single chemicals. Existing models for translation to population levels could be combined with eco-exposome assessment.

TK = toxicokinetics.

## Data Availability

Data are available from the corresponding author (stefan.scholz@ufz.de).

## TEXTBOX 1: Definition of the eco-exposome

The eco-exposome represents the totality of internal exposure over a lifetime to individuals of a given species. This includes exposure to anthropogenic chemicals, their biotransformation products, and/or adducts. Endogenous signaling chemicals, changed in response to exposure of anthropogenic chemicals, could contribute to the totality of an internal exposure and the translation of this exposure to biological responses.
